# A histopathological review of gynaecological malignancies in Katsina state North-Western Nigeria

**DOI:** 10.3332/ecancer.2024.1750

**Published:** 2024-08-29

**Authors:** Asma’u Usman, Shamsu Sahalu Bello, Aisha Abdurrahman, Fatima Abubakar Rasheed, Shuaibu Adam, Abubakar Dahiru

**Affiliations:** 1 Department of Anatomic and Molecular Pathology, Federal Teaching Hospital Katsina, Katsina 820101, Nigeria; 2 General Amadi Rimi Specialist Hospital Katsina, Katsina 820102, Nigeria; 3 Department of Obstetrics and Gynaecology, Federal Teaching Hospital Katsina, Katsina 820101, Nigeria

**Keywords:** histopathological, gynaecological malignancies, northwestern Nigeria, Katsina state

## Abstract

**Background:**

Gynaecological cancers, which affect the female genital tract, constitute a significant public health problem, especially in developing countries. Some of these malignancies have known aetiology and premalignant stages making them preventable. Understanding the burden of gynaecological malignancies in our environment will provide baseline information and help form strategies for their control.

**Aim:**

To describe the histological subtypes of gynaecological cancers, their frequency and age distribution trends in Katsina State over the 10-year study period.

**Methods:**

This was a 10-year retrospective cross-sectional multicenter study of all histologically diagnosed gynaecological cancer cases seen from 1st January 2012 to 31st December 2021 at Federal Teaching Hospital Katsina, General Hospital Katsina and General Amadi Rimi Specialist Hospital Katsina. Data for this study were extracted from departmental record registers of the pathology laboratories of the corresponding hospitals whose laboratories provide pathology services to the State. Cancer distribution over the years was sorted based on the primary site of diagnosis, histological diagnosis and age. Data were analysed using Statistical Package for Social Science version 28 and results were presented in tables and charts.

**Results:**

Two thousand three hundred and fifty-nine cancers were seen over the 10-year study period. Of these cases, 58.4% (*n* = 1,378) were females. Gynaecological malignancies accounted for 18.7% (441/2,359) of all cancers and 32.0% (441/1,378) of all female cancers. The highest frequency of gynaecological cancers was seen in women who were within the age groups of 40–49 and 50–59, and the lowest was seen in women who were ≥90 years old. The mean age was 48.9 ± 14.9 years. The most common site of gynaecological malignancies was the cervix uteri (*n* = 262, 59.4%) followed by the ovary (*n* = 106, 24.0%). Other sites in descending order were corpus uteri (*n* = 29, 6.6%), vulva (*n* = 9, 2.0%) and vagina (*n* = 2, 0.5%). The most common histo-morphologic subtypes were large-cell keratinizing squamous cell carcinoma in the cervix, large-cell non-keratinizing squamous cell carcinoma in the cervix and cystadenocarcinoma in the ovary. Choriocarcinoma was found in 33 cases (7.5%).

**Conclusion:**

This study demonstrated the various histotypes of gynaecological malignancies and their trends in Katsina state. The leading cancer was found to be cervical cancer which is mainly preventable. It is hoped that data from this study will provide a basis for making and implementing policies and strategies to lessen the problems of gynaecological malignancies through regular screening programs, especially for cervical cancer and accepting human papilloma virus (HPV) vaccination take-up.

## Introduction

Gynaecological cancers are malignancies that affect the female genital tract and are generally named according to the site of origin. They include cervical, uterine, ovarian, vulvar and vaginal cancers, and they collectively constitute a significant public health problem. These malignancies pose a considerable burden on the healthcare systems and the overall well-being of affected individuals. In addition, unlike malignancies elsewhere, gynaecological malignancies negatively impact fertility as they affect the reproductive system and their treatment often involves sterilizing surgery, chemotherapy or radiation. They affect millions of women worldwide, leading to substantial morbidity and mortality.

In 2020, gynaecological cancers accounted for 7.2% of all new cancer diagnoses and 6.8% of all cancer deaths globally [1]. The International Agency for Research on Cancer reported that gynaecological cancers globally contributed 19% of the 5.1 million estimated new cancer cases and 2.9 million cancer deaths [2]. In developing countries, approximately 1 in 4 of all cancers in women is gynaecological cancer due to the high incidence of cervical cancer, which accounts for up to 60% of the gynaecological cancer burden in these countries [3]. In Nigeria, cervical cancer is the second most common cancer diagnosed in women [4]. 

In developed countries, established preventive strategies, including human papilloma virus (HPV) vaccination and screening, have led to a drastic reduction in the incidence of cervical cancer, with ovarian and uterine cancers now becoming the most frequently diagnosed gynaecological cancers [5].

Gynaecological cancers can affect both children and adults, though they tend to be more common with advancing age and are more frequently diagnosed in postmenopausal women [6]. However, those diagnosed in women aged less than 25 years, including children, are more likely to be ovarian cancers, more specifically germ cell cancers [7].

Generally, cervical cancers and most vaginal and vulvar cancers have a known aetiology and recognized premalignant stages, making them potentially preventable through screening and treatment of premalignant lesions. Ovarian and uterine cancers, on the other hand, have no known infectious aetiology. Understanding the burden of these cancers is essential for developing strategies for the prevention and control of these malignancies, which will add to the existing literature on the burden of gynaecological cancers in Nigeria and serve as a baseline for future comparisons after interventions. This study was, therefore, conducted to determine the histopathological pattern and frequency of gynaecological cancers in Katsina state.

## Methodology

### Study design

It was a retrospective study.

### Site of study

The study was conducted at Federal Teaching Hospital Katsina (FTHKT), General Hospital Katsina (GHKT) and General Amadi Rimi Specialist Hospital Katsina (GARSH). Two hospitals – FTHKT and GHKT- are in Katsina Metropolis while GARSH is in Batagarawa local government area. They are the only hospitals with functional pathology departments that receive tissue specimens from all other hospitals in the state for histological diagnosis. Katsina State is situated in North-Western Nigeria at latitude 12.98553 and longitude 7.617144. Katsina has an estimated population of 7,831,319 and a land mass of 24,192 km^2^ [8].

FTHKT is a Tertiary Hospital that serves as a referral centre for secondary health care centres in Katsina. Gynaecological cancer cases are managed in the Department of Obstetrics and Gynaecology. The department has a Gynaecological oncology unit that manages patients with gynaecological cancers, together with the Oncology department. The hospital also has an Anatomic and Molecular Pathology Department where all cancer specimens biopsied or excised are sent for histological diagnosis.

GHKT is one of the largest secondary healthcare facilities in Katsina, and it has a high patient turnover. The hospital also has a Gynaecology unit, which manages cases of gynaecological cancers, and a Histopathology unit, which receives tissue biopsies and specimens for histopathological diagnosis.

GARSH is also a secondary healthcare facility in Katsina. The hospital does not have a Gynaecology unit, but it has a Histopathology unit that receives specimens from other health centres in the state. All these hospitals are located within a range of about 20 km from each other.

### Study population

The study population comprised all histologically confirmed gynaecological cancer cases from the three study sites from 1st January 2012 to the 31st December 2021.

### Eligibility criteria

All histopathologically confirmed gynaecological cancers were included in the study, provided all the information needed to complete the proforma was available in the records. Cases with incomplete records were excluded from the analysis.

### Source of data, type of data collected and data collection process

All cases diagnosed as various gynaecological cancers were extracted from the pathology departmental record registers and archived histopathology reports of the laboratories of these hospitals. Data were collected into preformed proformas, which included the primary site of the cancer, the histopathological type of the cancer, the patient's age and gender and the year of diagnosis.

### Data analysis and management

The data were entered into an Excel spreadsheet for cleaning, which was then transferred into Statistical Package for Social Sciences version 28 and analyzed. The data were summarized as frequencies and percentages and were presented in frequency tables, charts and photomicrographs.

### Ethical consideration

The Health Research Ethics Committees (HREC) of FTHKT and Katsina State Ministry of Health granted ethical approval for this study with HREC approval numbers FTHKT HREC no: 073 and Katsina HREC no: 719, respectively. Only the necessary data were collected from the records, and no patient identification was collected to maintain the anonymity of the data.

## Results

Over the 10-year study period, there were 2,359 histologically confirmed cancer cases. There were 443 cases of gynaecological cancers, two were excluded due to missing patient’s age in the records, and 441 were included in the final analysis, giving a retrieval rate of 99.5%.

**Figure 1.**
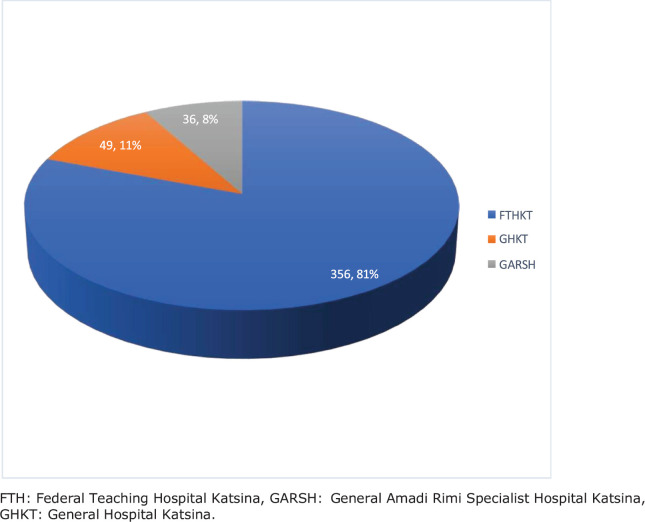
Pie chart illustrating the distribution of gynaecological malignancies according to hospital data in Katsina (2012–2021). FTH: Federal Teaching Hospital Katsina, GARSH: General Amadi Rimi Specialist Hospital Katsina, GHKT: General Hospital Katsina.

Of the 2,359 cancers that were seen, 1,378 (58.4%) were found in females. Gynaecological malignancies accounted for 18.7% (441/2,359) of all cancers and 32.0% (441/1,378) of all cancers in females. Among the gynaecological malignancies, 81.0% (*n* = 356) were seen in FTHKT, 11.0% (*n* = 49) in GHKT and 8.0% (*n* = 36) in GARSH Katsina. This is illustrated in Figure 1.

**Figure 2.**
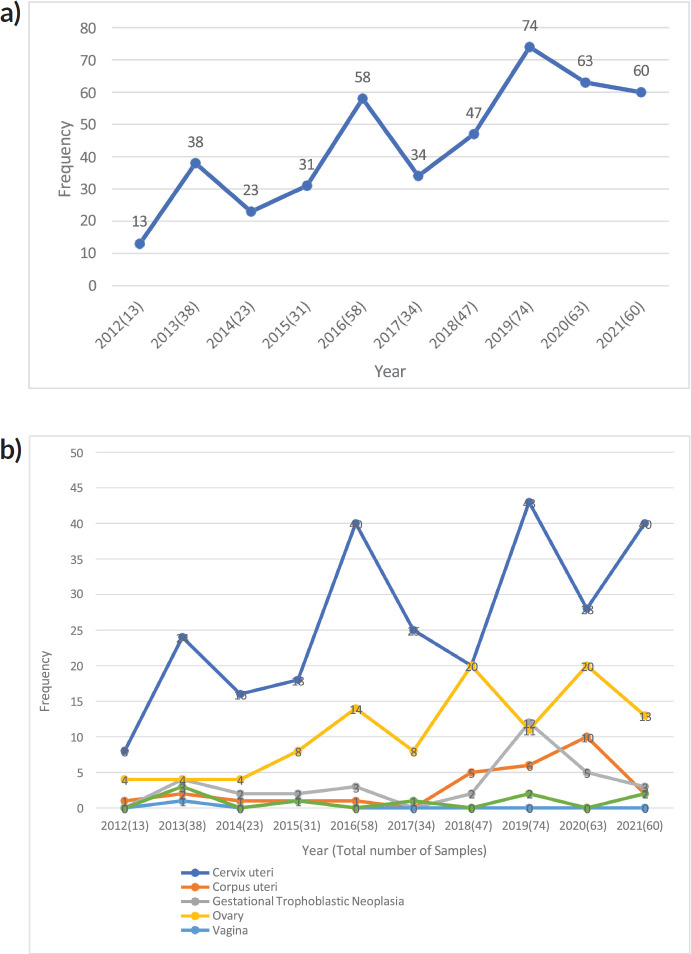
(a): Line graph showing the time-trend of all gynaecological malignancies in Katsina between 2012 and 2021. (b): Line graphs showing the distribution trend across the different types of gynaecological cancers in Katsina (2012–2021).

The distribution of all gynaecological malignancies over the years is shown in Figure 2a. There was a slow rise in cases from 13 in 2012 to 31 in 2015, a steep rise of 58 cases in 2016 and a sharp decline to 34 cases in 2017, possibly due to the numerous industrial disharmonies in the health sector that year. After that, it steadily rose to its peak of 74 cases in 2019, followed by a steady decline from 63 cases in 2020 to 60 cases in 2021, which may be attributed to the COVID-19 pandemic. The trend for each gynaecological malignancy is also shown in the Figure 2b. There was an increase in the number of cases of cervical and ovarian cancer diagnosed over the years. The rates of cancers of the corpus uteri and gestational trophoblastic neoplasia remained stable for the first 7 years reviewed and showed a slight rise in the last 3 years of the review. The rates of diagnosis of vaginal cancers however remained consistently low through the 10 years.

The distribution of malignancies based on the primary tissue site is seen in Table 1. The most common site of gynaecological malignancies was the cervix uteri (*n* = 262, 59.4%) followed by the ovary with 24% (*n* = 106). Other sites in descending fashion were gestational trophoblastic neoplasia (*n* = 33, 7.5%), corpus uteri (*n* = 29, 6.6%), vulva (*n* = 9, 2.0%) and vagina (*n* = 2, 0.5%).

**Figure 3.**
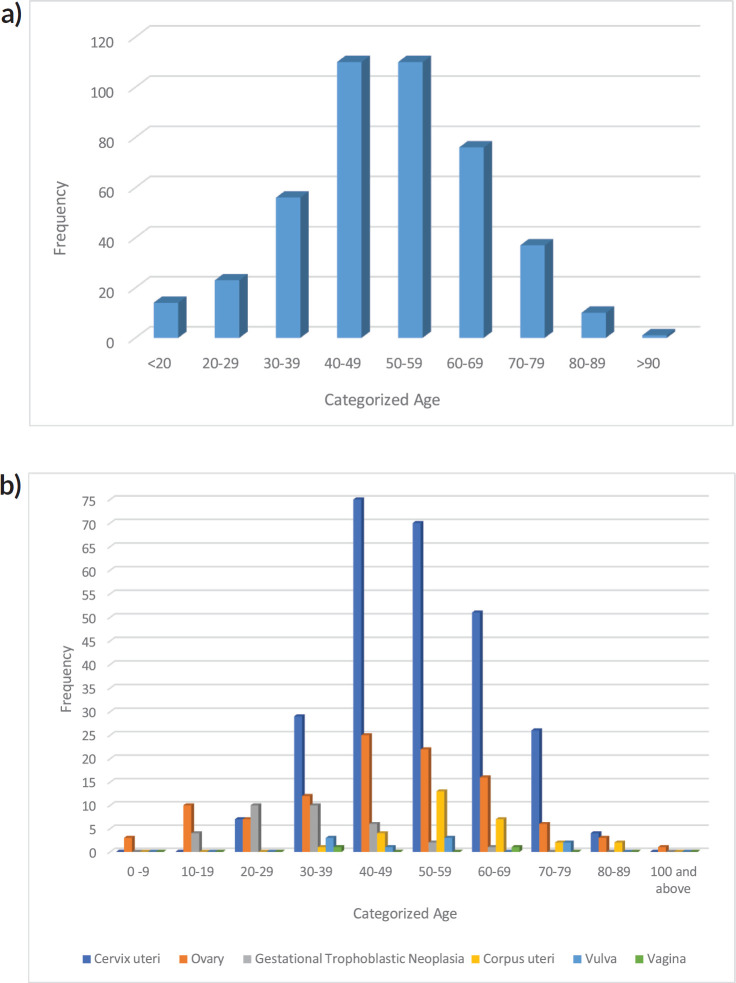
(a): Bar chart showing the categorized age distribution of total gynaecological malignancies in Katsina (2012–2021). (b): Bar chart showing categorized age distribution across the different types of gynaecological malignancies in Katsina, 2012–2021.

Figure 3a shows the age distribution of all gynaecological malignancies over the years of the study. The age of affected females ranged from 2 to 100 years with a mean age of 48.9 ± 14.9. The distribution is almost dome-shaped with the highest frequency seen in the 40–49 and 50–59 age groups and the lowest in the ≥90 years age group. The age distribution for each of the gynaecological malignancies is also shown in the Figure 3b. Cancers from the cervix and ovary had a peak in the 40–49 age group while corpus uteri cancer was seen more in the 50–59 age bracket. However, vulvar cancer had a bimodal peak and was seen within the 30–39 and the 50–59 age groups. Vaginal cancer was seen only in the 30–39 and 60–69 age groups.

The histological types of gynaecological malignancies based on the site of diagnosis are seen in Table 2. Being the most common gynaecological cancer, cervical cancer leads as the most frequent histo-morphologic type with 262 cases. Of the cervical cancer cases, large-cell keratinizing squamous cell carcinoma was the most common subtype (*n* = 121, 46.2%) followed closely by large-cell non-keratinizing squamous cell carcinoma (*n* = 105, 40.1%) and adenocarcinoma (*n* = 19, 7.2%) with leiomyosarcoma and adenoid cystic carcinoma as the least frequent (*n* = 1, 0.4%). Ovarian cancer was the second most common gynaecological malignancy, with 106 cases. Cystadenocarcinoma (*n* = 79, 74.2%) was the most occurring ovarian cancer subtype (serous type (40/79) more than mucinous type (37/79)) followed by yolk sac tumour (*n* = 10, 9.5%) and immature teratoma (*n* = 6, 5.7%). Dysgerminoma, granulosa cell tumour and malignant mixed mullerian tumour (MMMT) were diagnosed in 6 (5.7%), 2 (1.9%) and 2 (1.9%) cases, respectively. The least occurring ovarian cancer subtypes were malignant Brenner tumour, Burkitt and Hodgkin lymphomas, each with 1 case (1.0% each). Gestational trophoblastic neoplasia (choriocarcinoma) is the third most common, with 33 cases. This is followed closely by cancers of the corpus uteri with 29 cases and endometrioid carcinoma as its most frequent subtype (*n* = 14, 48.3%); vulvar cancer with 9 cases and squamous cell carcinoma as its most occurring subtype (*n* = 4, 44.5%); and vaginal cancer as the least common of all gynaecological cancers with only 2 cases.

**Figure 4.**
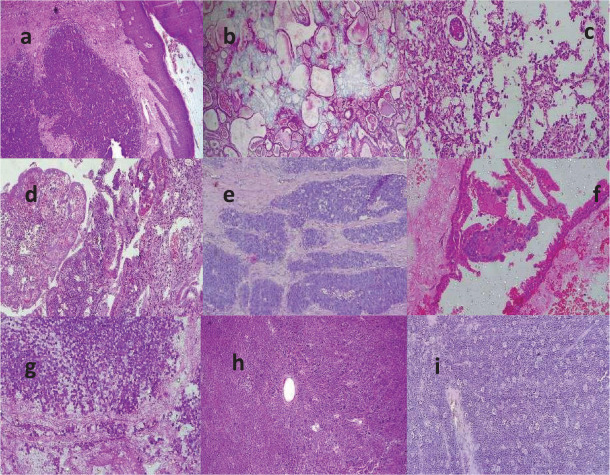
Photomicrographs showing histological sections of the gynaecological sites of diagnosis of malignancies in Katsina. (a): LCNKSCC of the cervix, H&E ×100, (b): Adenoid cystic carcinoma of the cervix, H&E ×100, (c): Serous papillary cystadenocarcinoma of the ovary, H&E ×200, (d): Adenosquamous carcinoma, H&E ×100, (e): Basal cell carcinoma of the vulva, H&E ×100, (f): Choriocarcinoma, H&E ×200, (g): Immature teratoma, H&E ×200, (h): Yolk sac tumour of ovary, H&E ×100 and (i): Ovarian Burkitt lymphoma, H&E ×100.

Figure 4 shows photomicrographs of histological sections of some of the gynaecological malignancies.

## Discussion

Gynaecological cancers have remained a major public health concern, especially in sub-Saharan Africa and a significant cause of morbidity and mortality in women worldwide. They are becoming the leading cause of gynaecologic-related deaths in developing countries, with variation in the distribution and frequency from one region to the other [2, 9, 10]. This is particularly so in developing countries with poor awareness and late presentation [10].

In this study, we looked at records over 10 years (2012–2021) where 441 gynaecological cancers were confirmed out of 2,359 histologically confirmed cancer cases in Katsina State over the study period. Gynaecological cancers contributed 18.7% of all cases of cancer in the state, which was similar to 17.6% in Owerri, (FUTHO) Nigeria [2]. The proportion was however higher when compared to data reported from other studies from Nigeria, where, 11.5% was reported from Aminu Kano Teaching Hospital [11], 10.1% in Enugu [12], 5.5% from the University of Abuja Teaching Hospital [13], 5.4% from Abakiliki and 3.6% reported from Ogbomosho, Nigeria. A lower value of 2.8% was seen in Ghana [14].

Cervical cancer, ranked first, was the most common gynaecological cancer seen, with 262 confirmed cases, which accounted for 59.4% of cases during the period of study. This finding was similar to the findings of 52.7%–62.9% reported by various studies across Nigeria [2, 13, 15–17]. However, it was slightly higher than the 48.6% seen in Kano [11], even though Kano state is in the same geopolitical zone. The higher proportion of cervical cancer in our studies when compared to other studies in our geopolitical zone may be due to a higher prevalence of risk factors for cervical cancer such as higher parity and cigarette smoking compared with other areas, as well as in lack of vaccination and poor screening. Katsina state has the highest fertility rate in Nigeria [18]. Again, a much higher frequency of 78% and 72.9% was reported from Enugu [12] and Edo [2], respectively.

Ovarian cancer was the second most common cancer in this study, accounting for 24.0% of cases. This figure is similar to 25.30% reported in Kano [11] as well as 20.9% in a multicenter review in the south-south and south-east regions [19] of Nigeria but higher than 7% reported in Lagos [20] despite ovarian cancer being their second commonest gynaecological cancer. The most common histological type was cystadenocarcinoma, which accounted for 16.9% of all the ovarian cancers in this study. This finding is similar to findings from Zaria [21], Maiduguri [16] and Lagos [20].

Choriocarcinoma accounts for 0.6% of all gynaecological cancers with the highest incidence rates seen in South-East Asia [22]. In this study, it is the third most common cancer, accounting for 7.5% of the cases. This finding is similar to findings in Kano, Nigeria [23] and Ghana, where 7.2% and 6.83% of their gynaecological malignancies were choriocarcinomas, respectively. In comparison, it was lower in Abuja, Nigeria (4.4%) [10] and South-Western Pakistan (4%) [24]. In Zaria [25] Nigeria, it was the most commonly occurring gynaecologic cancer but is the fourth most common in Ghana [14].

Globally, cancer of the uterine corpus is the sixth most commonly diagnosed [1]. The cancers that develop in the corpus uteri include endometrial carcinoma, endometrial stromal sarcoma, leiomyosarcoma and MMMT. Of these cancers, endometrial carcinoma is the most commonly diagnosed, with endometrial adenocarcinoma being the more predominant type [26]. However, in our study, cancer of the corpus uteri was the fourth most frequently diagnosed gynaecological malignancy, comprising 6.6% of all gynaecological malignancies. This finding is similar to studies conducted in Zaria [25] and Ilorin [15] in Nigeria and Mozambique [27], where cancer of the corpus uteri was not the most common gynaecological malignancy but rather the second, fourth and fifth, respectively. This finding contrasts with studies carried out in the USA [28] where it is the most common gynaecological malignancy. The histological types of corpus uteri cancers seen in this study correlate with the patterns observed in other studies in Nigeria [11, 29, 30].

Vulvar cancers accounted for 2.0% of overall gynaecological malignancies over the study period, ranking it as the fifth most frequent. The most common histological type was squamous cell carcinoma, followed by adenocarcinoma, while basal cell carcinoma and synovial sarcoma were the least diagnosed cancers, with one case each. Reports from various studies conducted in Nigeria and Mozambique showed a similar trend where vulvar cancers accounted for 1%–4% [12, 15, 20, 25] and 3.6% [27] of the total gynaecological malignancies. It was also found to be the fifth least anatomic site affected by cancer globally, with Europe and Asia having the highest burden, Africa having an intermediate burden, and Oceania having the lowest incidence [1].

Primary vaginal cancer is a rare cancer constituting 1%–2% of all malignancies [31]. Globally, in 2020, it accounted for 0.1% of all cancer cases [1]. It is the least common cancer in this study and is ranked 6^th^, accounting for 0.5% of all gynaecological malignancies. This finding is similar to what was seen in several studies in Nigeria [2, 10, 30] and the USA [32], where it accounted for 0.5%–2% of all female genital tract cancers. Contrastingly, studies from Ibadan [33] and Maiduguri, [34] Nigeria reported an incidence of 2% and 3.1%, respectively. The rarity of vaginal cancer is reaffirmed by a study from Enugu, Nigeria, where no case was seen over 10 years [12].

The limitations are that our study only reports on the histopathological pattern of cancer with the absence of other clinicopathological details such as the grade of cancer, stage of disease and immunohistochemical profile of some cancers, especially for therapeutic and prognostic purposes. However, the hospital has just been recently registered as a population-based cancer registry with the national cancer registry domiciled at the National Institute for Cancer Research and Training in Nigeria and thus, it is anticipated to project survival outcome data of patients’ and other variables.

## Conclusion

This multi-centred institutional-based study highlights the trend, pattern and histological type distributions of gynaecological malignancies in Katsina state. Cervical cancer was found to be the most common cancer and is largely preventable. It is anticipated that data from this study will provide a baseline for further studies that can be used in enacting policies and strategies to reduce gynaecological malignancies, especially with the establishment of a cancer registry through, among others, the implementation and domestication of screening programs for cervical cancer and the acceptance of HPV vaccination. Future research should include studies to evaluate the impact of the implementation of HPV vaccination and improved screening on the prevalence of cervical cancer in the state.

## Conflicts of interest

The authors declare that they have no conflict of interest concerning this study.

## Funding

None.

## Figures and Tables

**Table 1.** Distribution of malignancies based on primary site of pathology in Katsina (2012–2021).

S/N	Primary site	Frequency (*n*)	Percentage (%)
**1**	Cervix uteri	262	59.4
**2**	Ovary	106	24
**3**	Gestational trophoblastic neoplasia	33	7.5
**4**	Corpus uteri	29	6.6
**5**	Vulva	9	2
**6**	Vagina	2	0.5
	Total	441	100

**Table 2.** Histological subtypes based on site of diagnosis in Katsina (2012–2021).

S/N	Site	Frequency (*n*)	Percentage (%)
**I**	Cervix	262	100
	LCKSCC^a^	121	46.2
	LCNKSCC^b^	105	40.1
	Adenocarcinoma	19	7.2
	Adenosquamous	8	3.1
	Microinvasive squamous cell carcinoma	7	2.6
	Adenoid cystic carcinoma	1	0.4
	Leiomyosarcoma	1	0.4
**II**	Ovary	106	100
	Cystadenocarcinoma	79	74.2
	Yolk Sac tumour	10	9.5
	Immature teratoma	6	5.7
	Dysgerminoma	4	3.8
	Granulosa cell tumour	2	1.9
	Malignant mixed mullerian tumor	2	1.9
	Malignant brenner tumour	1	1
	Burkitt lymphoma	1	1
	Hodgkin lymphoma	1	1
**III**	Gestational trophoblastic neoplasia	33	100
	Choriocarcinoma	33	100
**IV**	Corpus uteri	29	100
	Endometrioid carcinoma	14	48.3
	Malignant mixed mullerian tumour	10	34.6
	Leiomyosarcoma	2	6.9
	Adenocarcinoma	1	3.4
	Serous carcinoma	1	3.4
	Endometrial stromal sarcoma	1	3.4
**V**	Vulva	9	100
	Squamous cell carcinoma	4	44.5
	Adenocarcinoma	3	33.3
	Basal cell carcinoma	1	11.1
	Synovial sarcoma	1	11.1
**V**	Vagina	2	100
	Clear cell carcinoma	1	50
	Adenocarcinoma	1	50

a LCKSS – Large-cell keratinizing squamous cell carcinoma,

b LCNKSCC – Large-cell non-keratinizing squamous cell carcinoma
